# Supplementary material for: EZH2 depletion potentiates MYC degradation inhibiting neuroblastoma and small cell carcinoma tumor formation
Source: Nat Commun. 2022 Jan 10;13:12. doi: 10.1038/s41467-021-27609-6 (PMC8748958; doi:10.1038/s41467-021-27609-6)
Supplement: Supplementary file 1 — Supplementary Information [file 41467_2021_27609_MOESM1_ESM.pdf]

Supplementary Information

EZH2 depletion potentiates MYC degradation inhibiting neuroblastoma and small cell carcinoma tumor formation

Liyuan Wang[1,2], Chan Chen[2], Zemin Song[3], Honghong Wang[3], Minghui Ye[2], Donghai Wang[2], Wenqian Kang[2], Hudan Liu[2], Guoliang Qing[1,2]*

[1]Department of Urology, Zhongnan Hospital of Wuhan University, Wuhan 430071, China

[2]Frontier Science Center for Immunology and Metabolism, Medical Research Institute, Wuhan University, Wuhan 430071, China

[3]Department of Pathophysiology, School of Basic Medical Sciences, Wuhan University, Wuhan 430071, China

*Correspondence: qingguoliang@whu.edu.cn

Supplementary Figure 1-7

Supplementary Table 1-2

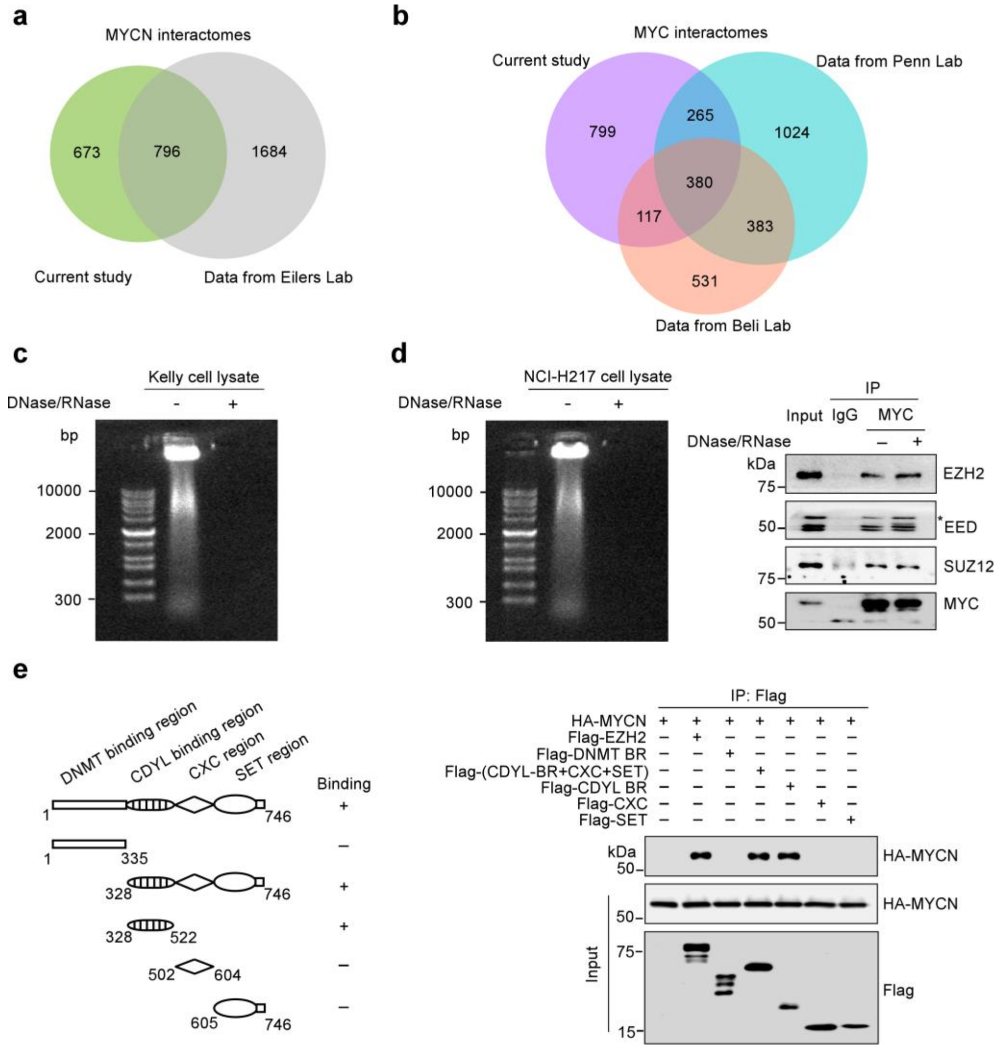

Supplementary Figure 1. Identification of EZH2 as a MYC(N) binding partner and characterization of minimal domain within EZH2 responsible for protein-protein interaction.

a Venn diagram of common MYCN-interacting partners between the current study and data from Eilers lab (Ref.13). **b** Venn diagram of overlapped MYC-interacting partners among current study and data from Beli and Penn labs (Ref.14 and 15). **c** Nucleic acid gel electrophoresis detection of lysates of Kelly cells treated with DNase/RNase (10 µg/mL) combination. **d** Co-immunoprecipitation (co-IP) to detect interaction between endogenous MYC and PRC2 components. Lysates of NCI-H2171 cells were treated with DNase/RNase (10 µg/mL) (left) and subjected to co-IP for detecting protein interaction (right). The asterisk denotes a nonspecific band. **e** Characterization the minimal domain within EZH2 for MYCN binding. Left: Schematic representation of EZH2 and various truncations; Right: 293T cells were transfected with plasmids expressing epitope-tagged MYCN, EZH2 or its truncations as shown. Anti-Flag associated precipitates were analyzed to detect the MYCN binding capacity with anti-HA antibody by immunoblot. DNMT-BR: DNA methyltransferase binding region; CDYL-BR: chromodomain Y-like protein binding region; CXC: cysteine-rich domain; SET: Su(var)3-9, enhancer of zeste, trithorax domain. The experiments were independently repeated three times with similar results (**c**-**e**). Source data are provided as a Source data file.

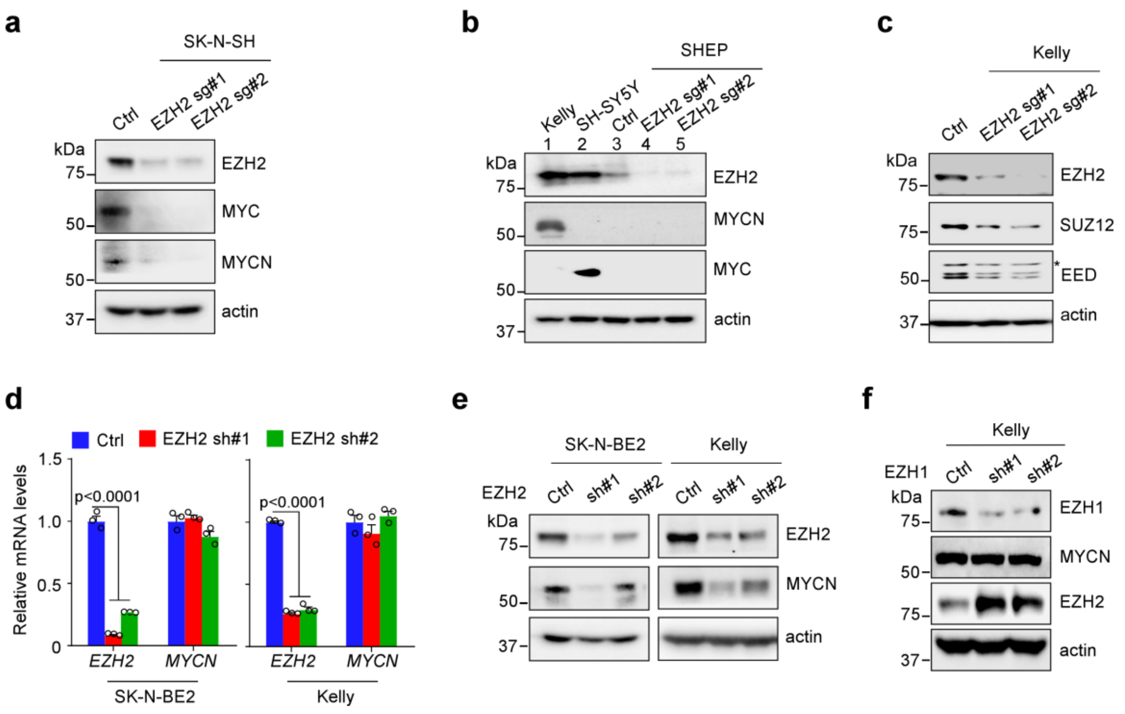

Supplementary Figure 2. Depletion of EZH2 selectively decreases MYC(N) protein levels.

a Immunoblot to detect MYCN and MYC levels upon EZH2 depletion with specific sgRNAs in SK-N-SH neuroblastoma cell lines. Actin was used as a loading control. **b** Comparison of MYCN and MYC protein levels in Kelly, SH-SY5Y and SHEP cells by immunoblot. EZH2 was depleted by specific sgRNAs as indicated. **c** Immunoblot assays to detect SUZ12 and EED levels upon EZH2 depletion with specific sgRNAs in Kelly cells. The asterisk denotes a nonspecific band. **d** Real-time qPCR analysis of *MYCN* mRNA levels in SK-N-BE2 and Kelly cells upon EZH2 depletion. Graph shows mean ± SD from triplicates, significance was determined by unpaired two-tailed Student's t test. **e** Immunoblot assays to detect MYCN levels upon EZH2 depletion with specific shRNAs in SK-N-BE2 and Kelly cells. **f** Immunoblot assays to detect MYCN and EZH2 levels upon EZH1 depletion in Kelly cells. The experiments were independently repeated three times with similar results (**a**-**c**, **e**-**f**). Source data are provided as a Source data file.

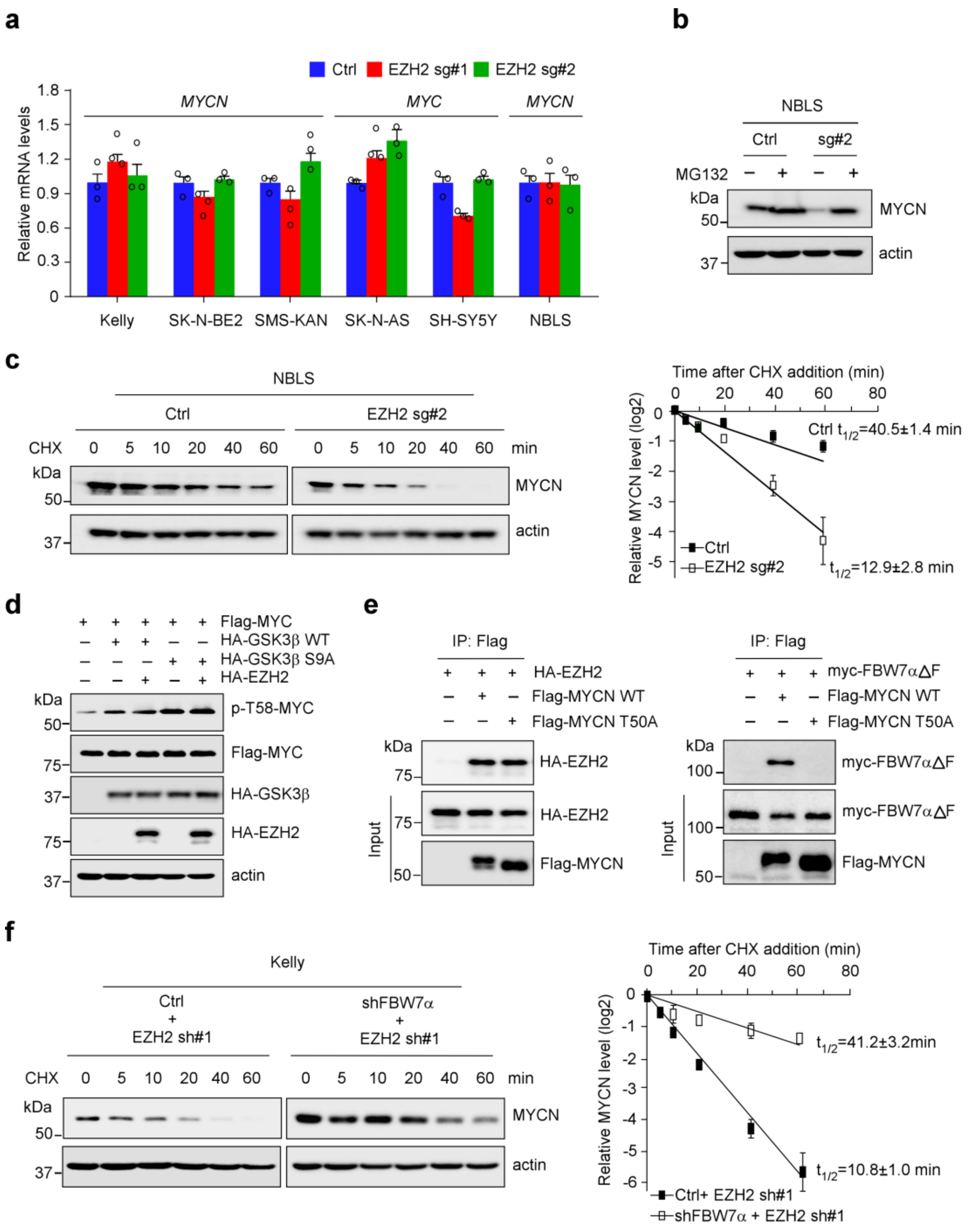

Supplementary Figure 3. EZH2 stabilizes MYCN by counteracting FBW7α–mediated polyubiquitination.

a Real-time qPCR analysis of *MYCN* or *MYC* mRNA levels upon EZH2 depletion by specific sgRNAs in *MYCN*-amplified (Kelly, SK-N-BE2 and SMS-KAN) and *MYCN*-nonamplified (SK-N-AS, SH-SY5Y and NBLS) neuroblastoma cells. Graph shows mean ± SD from biological triplicates, significance was determined by unpaired two-tailed Student's t test. **b** Immunoblots of MYCN in NBLS cells with or without EZH2 depletion. Cells were treated with MG132 (5 µM) for 6 hr before harvest as indicated. **c** Time-course analysis of MYCN protein levels in EZH2-depleted NBLS cells from one representative experiment (left). MYCN band

density relative to actin was quantified and plotted on the right. Data shown were obtained from averages of three independent experiments. **d** Analysis of MYC phosphorylation status in the presence or absence of EZH2. 293T cells were transfected with plasmids expressing MYC, GSK3β WT or kinase-active mutant (S9A), and EZH2 for 48 hr as indicated. MYC Phosphorylation at the threonine 58 (T58) (p-T58-MYC) was detected by immunoblot. **e** Co-IP to detect protein-protein interaction between EZH2 and MYCN (left), or FBW7αΔF and MYCN (right). 293T cells were transfected with plasmids expressing epitope-tagged MYCN (WT or T50A), EZH2 or FBW7αΔF for 48 hr as shown. Anti-Flag associated precipitates were analyzed for protein-protein interaction by immunoblot with anti-HA and anti-myc antibodies, respectively. **f** Time-course analysis of MYCN protein levels in EZH2-depleted Kelly cells with or without FBW7α depletion from one representative experiment (left). MYCN band density relative to actin was quantified and plotted on the right. Immunoblots show one representative result. The experiments were independently repeated three times with similar results (**b**, **d**-**e**). Source data are provided as a Source data file.

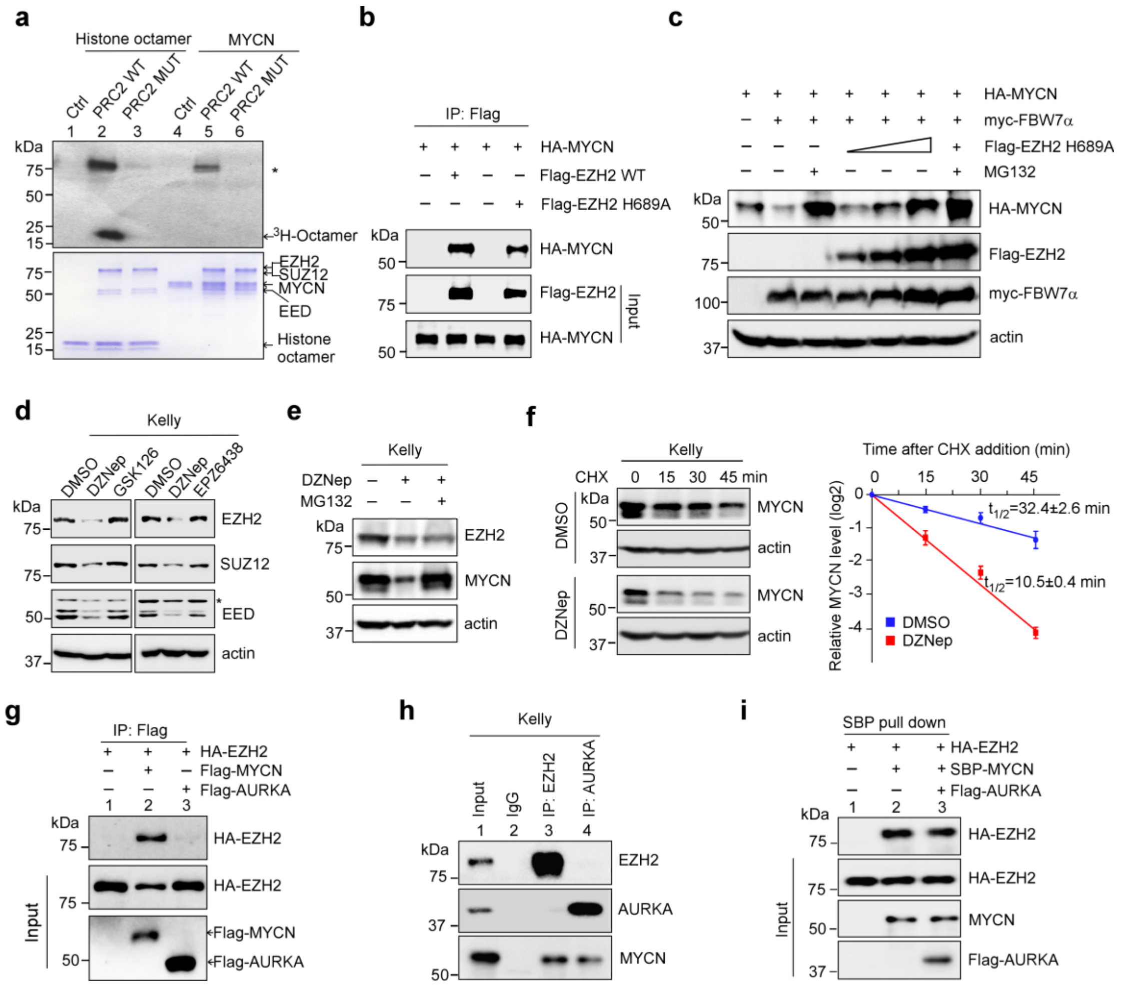

Supplementary Figure 4. EZH2 promotes MYCN stabilization independent of its methyltransferase activity.

a Analysis of MYCN methylation. *In vitro* methylation assay was performed using recombinant MYCN in the presence of purified polycomb repressive complex 2 (PRC2) containing EZH2 WT or H689A mutant (MUT). Recombinant histone octamer was used as a positive control. Loading controls were shown as Coomassie blue staining in the bottom panel. **b** Detection of MYCN interaction with EZH2 WT or H689Amutant. 293T cells were transfected with plasmids expressing epitope-tagged MYCN and EZH2 WT or H689A mutant as shown. Anti-Flag associated precipitates were used for MYCN binding assays by immunoblot with anti-HA antibody. **c** Analysis of MYCN abundance with increasing doses of EZH2 H689A mutant. 293T cells were transfected with plasmids expressing MYCN, FBW7α and increasing amounts of EZH2 H689A mutant for 48 hr as shown. MYCN and other indicated proteins were analyzed by immunoblot. **d** Immunoblots of EZH2, SUZ12 and EED in the presence of various EZH2 inhibitors. Kelly cells were respectively treated with 5 μM of DZNep, GSK126 or EPZ6438 for 48 hr before subjected to immunoblotting. The asterisk denotes a nonspecific band. **e** Immunoblots of MYCN in Kelly cells in the presence of DZNep and/or MG132. Cells were treated with DZNep (5 μM) for 48 hr and subjected to MG132 (5 μM) for 6 hr before harvest as indicated. **f** Time-course analysis of MYCN protein levels in Kelly cells treated with DZNep (5 μM) from one representative experiment (left). MYCN band density relative to actin was

quantified and plotted on the right. Data shown were obtained from averages of three independent experiments. **g** Analysis of interaction between EZH2 and AURKA. 293T cells were transfected with plasmids expressing epitope-tagged EZH2 and AURKA or MYCN for 48 hr as shown. Anti-Flag associated precipitates were analyzed to detect the EZH2-AURKA or EZH2-MYCN interaction. The EZH2 -MYCN interaction was used as a positive control. **h** Co-IP to detect interaction between endogenous EZH2 and AURKA. Lysates from Kelly cells were subjected to immunoprecipitation using antibodies against EZH2 or AURKA, and then were analyzed by immunoblot with anti-EZH2, -AURKA and -MYCN antibodies, respectively. **i** EZH2 and MYCN interaction in the presence or absence of AURKA. 293T cells were transfected with plasmids expressing epitope-tagged MYCN, EZH2 and AURKA for 48 hr as shown. SBP pull-down was performed and associated precipitates were analyzed for EZH2 binding by immunoblot with anti-HA antibody. The experiments were independently repeated three times with similar results (**a-e**, **g-i**). Source data are provided as a Source data file.

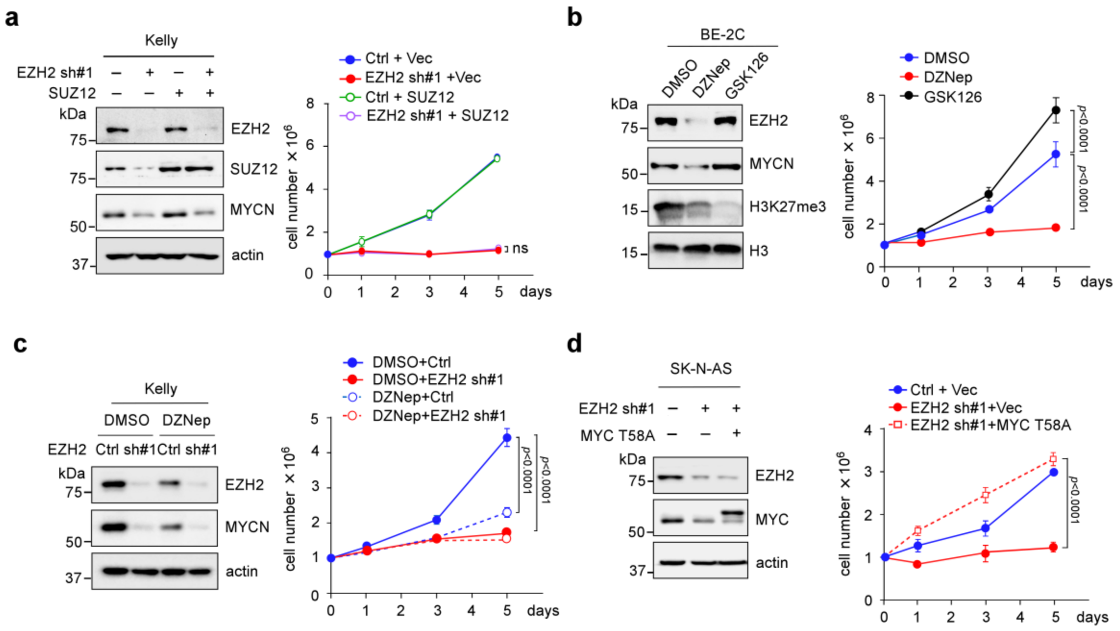

Supplementary Figure 5. Depletion of EZH2 as a whole, but not its enzymatic inactivation, reduces MYCN expression and suppresses neuroblastoma tumor cell growth.

a Immunoblot of MYCN (left) and proliferation analysis (right) of Kelly cells upon shRNA depletion of endogenous EZH2 in the presence or absence of ectopically expressed SUZ12. **b** Immunoblot of MYCN (left) and proliferation analysis (right) of BE-2C cells which were respectively treated with 5 μM of DZNep or GSK126 for 48 hr. **c** Immunoblot of MYCN (left) and proliferation analysis (right) of Kelly cells upon shRNA depletion of endogenous EZH2 with or without DZNep treatment. **d** Immunoblots of EZH2 and MYC in SK-N-AS cells which were treated with DZNep (5 μM) with or without ectopic expression of the MYC T58A mutant (left). Cell proliferation was plotted on the right. Graph shows mean ± SD from biological triplicates, significance was determined by two-way (**a, b, c** and **d**) ANOVA test followed by Tukey's correction. Source data are provided as a Source data file.

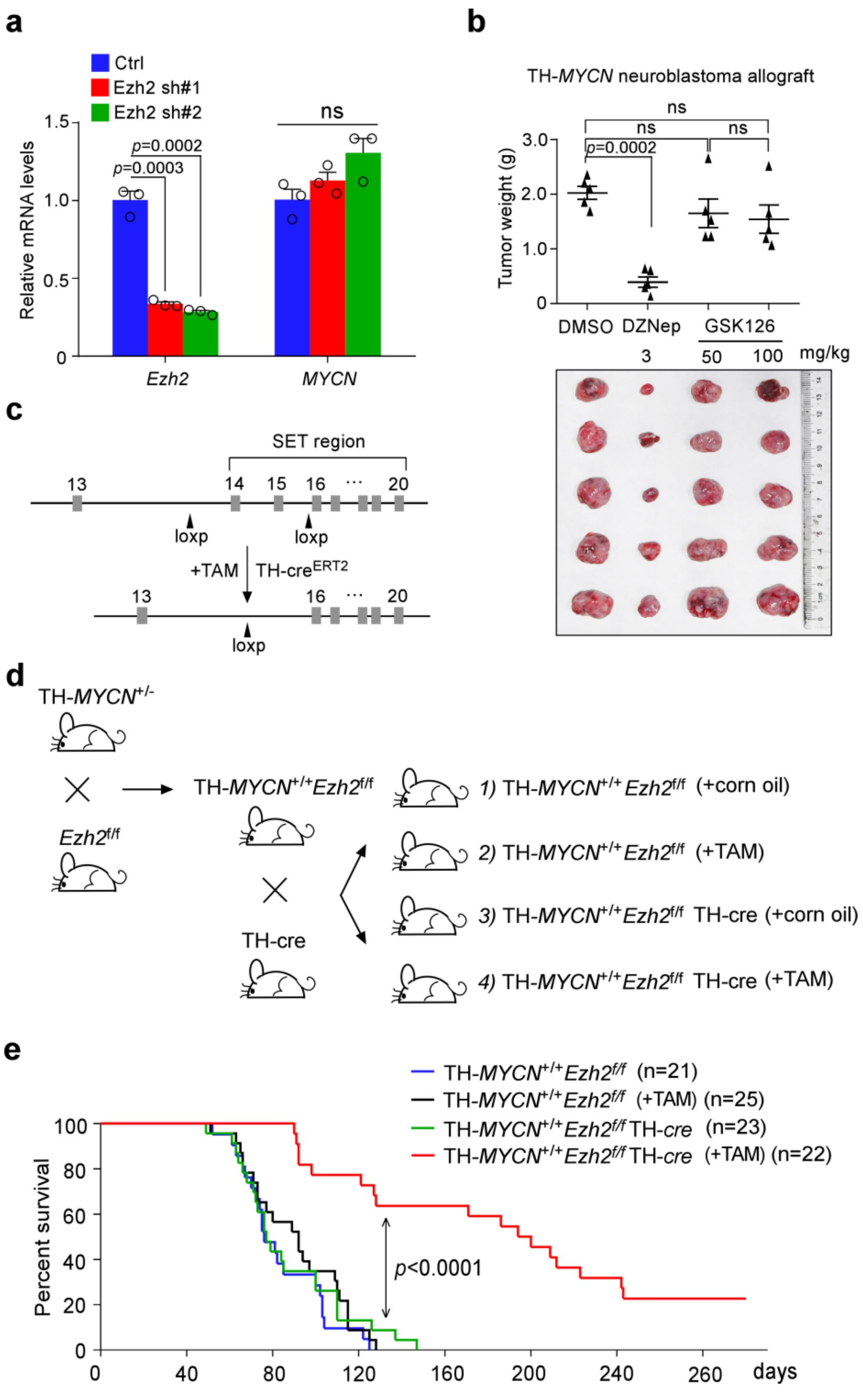

Supplementary Figure 6. Pharmacological or genetic inhibition of EZH2 inhibits neuroblastoma tumor growth in vivo.

a Real-time qPCR analysis of *MYCN* mRNA level upon *Ezh2* depletion in primary neuroblastoma tumor cells obtained from TH-*MYCN*$^{+/+}$ mice. Graph shows mean ± SD from biological triplicates, significance was determined by unpaired two-tailed Student's t test. **b** TH-*MYCN* neuroblastoma allograft mice were subjected to DZNep or GSK126 treatment at the indicated doses. Images of subcutaneous tumors (n=5 per group) are shown on the bottom. Graph shows mean ± SD, significance was determined by one-way ANOVA test followed by Tukey's correction. **c** Targeting strategy to generate *Ezh2* conditional knockout alleles. Boxes

represent exons and lines represent introns. Exon number is shown above each box. **d** Breeding scheme for conditional knockout of *Ezh2* alleles in TH-*MYCN* mice. When needed, tamoxifen (TAM, 100 mg/kg, resolved in corn oil) was intraperitoneally injected once daily for five consecutive days to achieve conditional *Ezh2* knockout. **e** Kaplan-Meier survival plot of *Ezh2*-deleted TH-*MYCN*$^{+/+}$ mice and age-matched controls. Significance was determined by logrank test. Source data are provided as a Source data file.

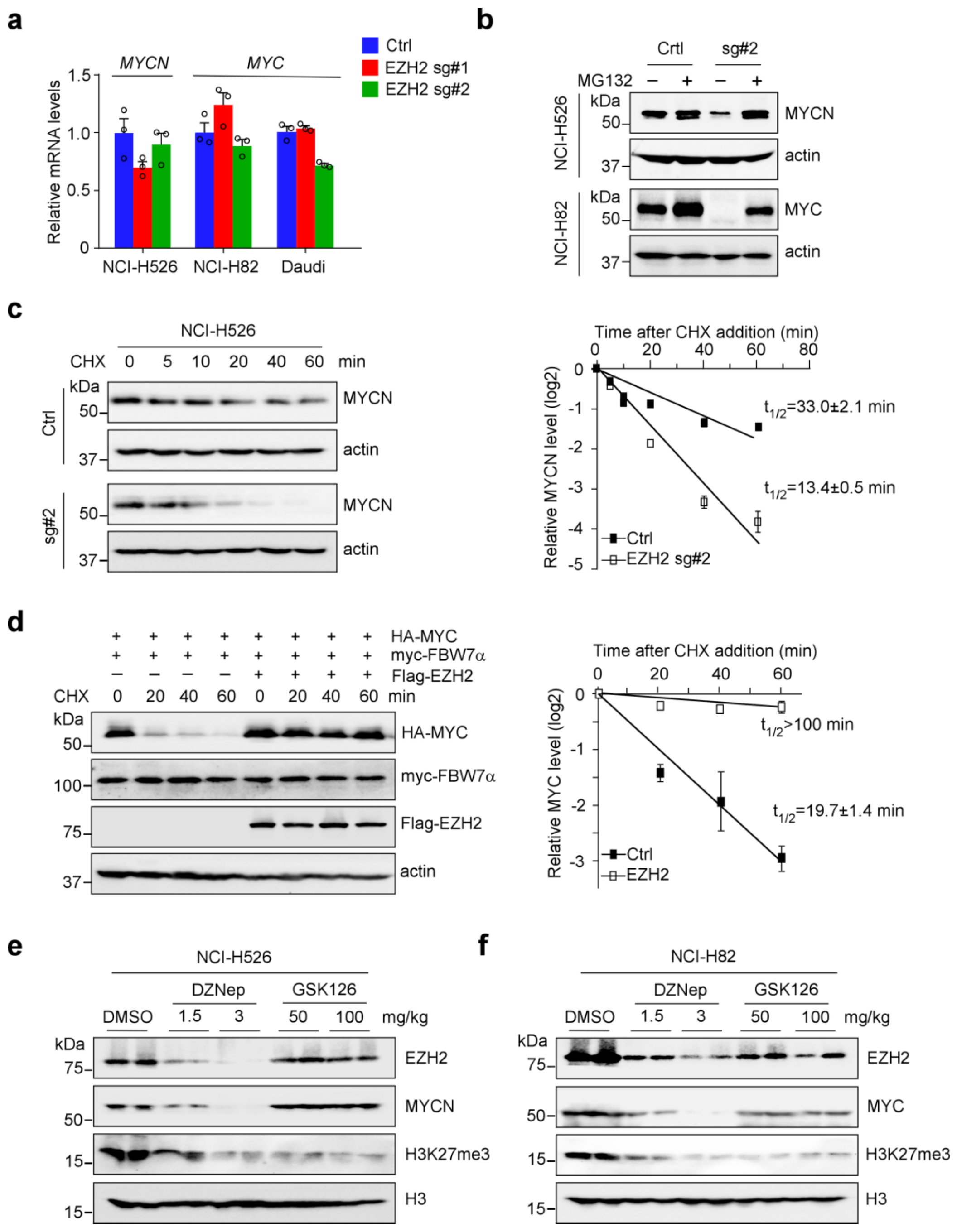

Supplementary Figure 7. EZH2 depletion induces MYC(N) degradation in small cell lung carcinoma and Burkitt's lymphoma cells.

a Real-time qPCR analysis of *MYCN* and *MYC* mRNA levels upon EZH2 depletion by specific sgRNAs in NCI-H526, NCI-H82 and Daudi cells as indicated. Graph shows mean ± SD from biological triplicates, significance was determined by unpaired two-tailed Student's t test. **b** Immunoblots of MYCN and MYC in NCI-H526 and NCI-H82 cells with or without EZH2 depletion. Cells were treated with MG132 (5 μM) for 6 hr before harvest as indicated. **c** Time-course analysis of MYCN protein levels in EZH2-depleted NCI-H526 cells from one representative experiment (left). MYCN band density relative to actin was quantified and

plotted on the right. Data shown were obtained from averages of three independent experiments. **d** Time-course analysis of HA-MYC levels in 293T cells expressing ectopic HA-MYC, myc-FBW7α, and/or Flag-EZH2 as indicated (left). MYC band density relative to actin was quantified and plotted on the right. Data shown are means (±SD) of three independent experiments. **e,f** NCI-H526 or NCI-H82 xenografts were subjected to DZNep or GSK126 treatment at the indicated doses. Immunoblots of indicated proteins from representative NCI-H526 (**e**) or NCI-H82 (**f**) xenograft tumors are presented. The experiments were independently repeated three times with similar results (**b**, **e**-**f**). Source data are provided as a Source data file.

Supplementary Table 1. A list of antibodies used in the study.

Target	Supplier	Catalog No.	Application/ Dilution or amount
actin	ABclonal	AC026	WB/ 1:5000
AURKA	Cell Signaling Technology	14475	WB/ 1:1000<br>IP/ 1 μg
Cad	Proteintech	16617-1-AP	WB/ 1:1000
EED	Cell Signaling Technology	85322	WB/ 1:1000
EZH1	Cell Signaling Technology	42088	WB/ 1:1000
EZH2	Cell Signaling Technology	5246	WB/ 1:1000<br>IP/ 1μg<br>IHC/ 1:500
FBW7α	Abcam	ab109617	WB/ 1:1000
Flag-tag	Sigma-Aldrich	F1804	WB/ 1:1000<br>IP/ 1 μg
HA-tag	ABclonal	AE008	WB/ 1:1000<br>IP/ 1 μg
HA-tag-HRP	Roche	1201381900	WB/ 1:1000
HUWE1	Proteintech	19430-1-AP	WB/ 1:1000
Histone H3	Abcam	ab176842	WB/ 1:3000
Ldha	Cell Signaling Technology	2012	WB/ 1:1000
MYC	Santa Cruz Biotechnology	sc-764	WB/ 1:1000<br>IP/ 1 μg
MYC	Cell Signaling Technology	13987	WB/ 1:1000
myc-tag	ABclonal	AE010	WB/ 1:1000<br>IP/ 1 μg
MYCN	Santa Cruz Biotechnology	sc-53993	WB/ 1:1000<br>IP/ 1 μg<br>ChIP/ 5 μg<br>IHC/ 1:200
p-T58-MYC	Santa Cruz Biotechnology	sc-135647	WB/ 1:1000
Pol II	Cell Signaling Technology	14958	ChIP/ 5 μg
SUZ12	Abcam	ab12073	WB/ 1:1000
H3K27me3	Abcam	ab 6002	WB/ 1:3000<br>IHC/ 1:800
Ubi	ABclonal	A3207	WB/ 1:1000
Anti-Rabbit IgG	Jackson ImmunoResearch	JAC-111-035-003	WB/ 1:10000
Anti-Mouse IgG	Jackson ImmunoResearch	JAC-115-035-003	WB/ 1:10000

Supplementary Table 2. Primers and shRNAs/sgRNAs used in this study.

**Primers**	
**Primers for real-time PCR**	
Human *ACTIN* forward	5′-CACCATTGGCAATGAGCGGTTC-3′
Human *ACTIN* reverse	5′-AGGTCTTTGCGGATGTCCACGT-3′
Human *EZH2* forward	5′-GACCTCTGTCTTACTTGTGGAGC-3′
Human *EZH2* reverse	5′-CGTCAGATGGTGCCAGCAATAG-3′
Human *MCT1* forward	5′-TTGTTGGTGGCTGCTTGTCAGG-3′
Human *MCT1* reverse	5′-TCATGGTCAGAGCTGGATTCAAG-3′
Human *MYC* forward	5′-CTGGTGCTCCATGAGGAGA -3′
Human *MYC* reverse	5′-CCTGCCTCTTTTCCACAGAA-3′
Human *MYCN* forward	5′-CCACAAGGCCCTCAGTACC-3′
Human *MYCN* reverse	5′-TCTTCCTCTTCATCATCTTCATC-3′
Human *NCL* forward	5′-CCCCAGAACCAAAATGGCAAA-3′
Human *NCL* reverse	5′-AGAGTTTTGGATGGCTGGCTT-3′
Human *ODC* forward	5′-CCAAAGCAGTCTGTCGTCTCAG-3′
Human *ODC* reverse	5′-CAGAGATTGCCTGCACGAAGGT-3′
Human *PRMT5* forward	5′-CTAGACCGAGTACCAGAAGAGG-3′
Human *PRMT5* reverse	5′-CAGCATACAGCTTTATCCGCCG-3′
Human *SRSF1* forward	5′-ATGTCGGGAGGTGGTGTGATTC-3′
Human *SRSF1* reverse	5′-TGTTCCACGGCCGCTTCGAG-3′
Human *YWHAZ* forward	5′-CCTCACTCCCGTTTCCG-3′
Human *YWHAZ* reverse	5′-CAGCACCTTCCGTCTTT-3′
**Primers for ChIP assay**	
ACTIN promoter forward	5'-GACTTCTAAGTGGCCGCAAG-3'
ACTIN promoter reverse	5'-TTGCCGACTTCAGAGCAAC-3'
NCL promoter forward	5'-CTCGGGGTGGAGAGATGAGA-3'
NCL promoter reverse	5'-GACTCCGACTAGGGCCGATA-3'
PRMT5 promoter forward	5'-GGACCCCGCATTCC-3'
PRMT5 promoter reverse	5'-GACAGCGCGAGGAGA-3'
**Primers for genotyping**	
TH-*MYCN* mice	
Chr18F1	5′-ACTAATTCTCCTCTCTCTGCCAGTATTTGC-3′
Chr18R2	5′-TGCCTTATCCAAAATATAAATGCCCAGCAG-3′
OUT1	5′-TTGGCACACACAAATGTATATACACAATGG-3′
Ezh2$^{f/f}$ mice	
primer forward	5′-CATGTGCAGCTTTCTGTTCA-3′
primer reverse	5′-CACAGCCTTTCTGCTCACTG-3′
TH-*cre*^{ERT2} mice	
primer1	5′-CGCATAGAAATTGCATCAACGCAT-3′
Primer2	5′-AGTTTGACCCGTACACCCTGGCCAT-3′
Primer3	5′-GGAGACCTTTCCTTCCTTTATTGAGA-3′

**shRNA and sgRNA sequences**	
shEZH2#1 (3’UTR)	5′- GAAACAGCTGCCTTAGCTTCA -3′
shEZH2#2	5′-CGGAAATCTTAAACCAAGAAT-3′
shEZH1#1	5′-TGGATGACTTATGCGTGATTT-3′
shEZH1#2	5′-AGACGTGCAAGCAGGTCTTTC-3′
shFBW7α	5′-TGATACATCAATCCGTGTTTG-3′
shHUWE1	5′-CGACGAGAACTAGCACAGAAT-3′
sgEZH2#1	5′-GGTCCCAATTAACCTAGCAA-3
sgEZH2#2	5′-ACAGAAGTCAGGATGTGCAC-3′
