# Supplementary material for: EZH2 depletion potentiates MYC degradation inhibiting neuroblastoma and small cell carcinoma tumor formation
Source: Nat Commun. 2022 Jan 10;13:12. doi: 10.1038/s41467-021-27609-6 (PMC8748958; doi:10.1038/s41467-021-27609-6)
Supplement: Supplementary file 3 — Description of Additional Supplementary Files [file 41467_2021_27609_MOESM3_ESM.pdf]

Description of Additional Supplementary Files

File Name: Supplementary Data 1
Description: MYCN-associated proteins by mass spectrometry analysis. Data shown were obtained from averages of three independent experiments. Significance was determined by unpaired two-tailed Student‟s t test

File Name: Supplementary Data 2
Description: MYC-associated proteins in mass spectrometry analysis. Data shown were obtained from averages of three independent experiments. Significance was determined by unpaired two-tailed Student‟s t test
