# Supplementary material for: EZH2 depletion potentiates MYC degradation inhibiting neuroblastoma and small cell carcinoma tumor formation
Source: Nat Commun. 2022 Jan 10;13:12. doi: 10.1038/s41467-021-27609-6 (PMC8748958; doi:10.1038/s41467-021-27609-6)
Supplement: Supplementary file 6 — Reporting Summary [file 41467_2021_27609_MOESM6_ESM.pdf]

nature portfolio

Corresponding author(s): Guoliang Qing
Last updated by author(s): Nov 11, 2021

Reporting Summary

Nature Portfolio wishes to improve the reproducibility of the work that we publish. This form provides structure for consistency and transparency in reporting. For further information on Nature Portfolio policies, see our Editorial Policies and the Editorial Policy Checklist.

Statistics

For all statistical analyses, confirm that the following items are present in the figure legend, table legend, main text, or Methods section.

n/a	Confirmed	
☐	☒	The exact sample size (*n*) for each experimental group/condition, given as a discrete number and unit of measurement
☐	☒	A statement on whether measurements were taken from distinct samples or whether the same sample was measured repeatedly
☐	☒	The statistical test(s) used AND whether they are one- or two-sided<br>*Only common tests should be described solely by name; describe more complex techniques in the Methods section.*
☒	☐	A description of all covariates tested
☐	☒	A description of any assumptions or corrections, such as tests of normality and adjustment for multiple comparisons
☐	☒	A full description of the statistical parameters including central tendency (e.g. means) or other basic estimates (e.g. regression coefficient) AND variation (e.g. standard deviation) or associated estimates of uncertainty (e.g. confidence intervals)
☐	☒	For null hypothesis testing, the test statistic (e.g. *F*, *t*, *r*) with confidence intervals, effect sizes, degrees of freedom and *P* value noted<br>*Give P values as exact values whenever suitable.*
☒	☐	For Bayesian analysis, information on the choice of priors and Markov chain Monte Carlo settings
☒	☐	For hierarchical and complex designs, identification of the appropriate level for tests and full reporting of outcomes
☒	☐	Estimates of effect sizes (e.g. Cohen's *d*, Pearson's *r*), indicating how they were calculated

Our web collection on statistics for biologists contains articles on many of the points above.

Software and code

Policy information about availability of computer code

Data collection	The qRT-PCR data: Bio-Rad CFX96 (Version 3.1)<br>The mass spectrometry data: Thermo Fisher Scientific Orbitrap Exploris 480 mass spectrometer<br>The western blot imaging: Bio-Rad Chemi DocTM Touch Imaging System
Data analysis	Statistical analysis: Graph Pad Prism (ver. 7)<br>qRT-PCR analysis: B10-Rad CFX Manager (Version 3.1)<br>Western Blot quantitation: ImageJ (Versionl.8.0, developed by NIH)<br>PET images display: Carimas (version: 1.05.210331)

For manuscripts utilizing custom algorithms or software that are central to the research but not yet described in published literature, software must be made available to editors and reviewers. We strongly encourage code deposition in a community repository (e.g. GitHub). See the Nature Portfolio guidelines for submitting code & software for further information.

Data

Policy information about availability of data

All manuscripts must include a data availability statement. This statement should provide the following information, where applicable:

- Accession codes, unique identifiers, or web links for publicly available datasets
- A description of any restrictions on data availability
- For clinical datasets or third party data, please ensure that the statement adheres to our policy

The mass spectrometry proteomics data GENERATED in this study have been deposited in the ProteomeXchange database under accession code PXD029652. The ChIP-seq data GENERATED in this study have been deposited in the NCBI database under accession code GSE181782 (https://www.ncbi.nlm.nih.gov/geo/query/

acc.cgi?acc=GSE181782). The remaining data are available within the Article, Supplementary Information or Source Data file.

Field-specific reporting

Please select the one below that is the best fit for your research. If you are not sure, read the appropriate sections before making your selection.

- [x] Life sciences
- [ ] Behavioural & social sciences
- [ ] Ecological, evolutionary & environmental sciences

For a reference copy of the document with all sections, see nature.com/documents/nr-reporting-summary-flat.pdf

Life sciences study design

All studies must disclose on these points even when the disclosure is negative.

Sample size	For in vitro experiments, at least three biologically independent experiments were performed for all experiments unless otherwise stated. No statistical method was used to predetermine sample size. Such sample sizes are typical for the in vitro experiments and sufficient for a statistical analysis. For in vivo experiments, a sample size of n = 5-6 mice per group were used, which is sufficient to generate statistically significant results. No statistical method was used to predetermine sample size.
Data exclusions	No data were excluded throughout the studies.
Replication	The replication numbers were described in the corresponding figure legends.
Randomization	For in vitro experiments, cells were randomly allocated into control and experimental groups. For in vivo experiments, age matched mice were randomized into all experimental groups.
Blinding	Data collection of mouse tumor experiments were performed in a double blinding manner, the investigators were blinded to group allocation during 'data analysis' of mouse tumor experiments. Cellular and biochemical experiments were not performed in a blinding manner because the same investigator was doing group allocation during data collection and/or analysis. For other experiments, investigators were not blinded to the identity of samples to ensure appropriate data collection and because experimental results are quantitative in nature, not readily subject to investigator bias. To ensure consistent experimental conditions, all control and experimental samples were processed in parallel.

Reporting for specific materials, systems and methods

We require information from authors about some types of materials, experimental systems and methods used in many studies. Here, indicate whether each material, system or method listed is relevant to your study. If you are not sure if a list item applies to your research, read the appropriate section before selecting a response.

Materials & experimental systems

n/a	Involved in the study	
[ ]	[x]	Antibodies
[ ]	[x]	Eukaryotic cell lines
[x]	[ ]	Palaeontology and archaeology
[ ]	[x]	Animals and other organisms
[x]	[ ]	Human research participants
[x]	[ ]	Clinical data
[x]	[ ]	Dual use research of concern

Methods

n/a	Involved in the study	
[ ]	[x]	ChIP-seq
[x]	[ ]	Flow cytometry
[x]	[ ]	MRI-based neuroimaging

Antibodies

Antibodies used

Target,Supplier,Catalog No.,Application/ Dilution or amount
actin,ABclonal,AC026,WB/ 1:5000
AURKA,Cell Signaling Technology,14475,WB/ 1:1000,IP/ 1 ug
Cad,Proteintech,16617-1-AP,WB/ 1:1000
EED,Cell Signaling Technology,85322,WB/ 1:1000
EZH1,Cell Signaling Technology,42088,WB/ 1:1000
EZH2,Cell Signaling Technology,5246,WB/ 1:1000,IP/ 1ug,IHC/ 1:500
FBW7a,Abcam,ab109617,WB/ 1:1000
Flag-tag,Sigma-Aldrich,F1804,WB/ 1:1000,IP/ 1 ug
HA-tag,ABclonal,AE008,WB/ 1:1000,IP/ 1 ug
HA-tag-HRP,Roche,1201381900,WB/ 1:1000
HUWE1,Proteintech,19430-1-AP,WB/ 1:1000
Histone H3,Abcam,ab176842,WB/ 1:3000
Ldha,Cell Signaling Technology,2012,WB/ 1:1000

	MYC,Santa Cruz Biotechnology,sc-764 WB/ 1:1000,IP/ 1 ug<br>MYC,Cell Signaling Technology,13987,WB/ 1:1000<br>myc-tag,ABclonal,AE010,WB/ 1:1000,IP/ 1 ug<br>MYCN,Santa Cruz Biotechnology,sc-53993 WB/ 1:1000,IP/ 1 ug,ChIP/ 5 ug,IHC/ 1:200<br>p-T58-MYC,Santa Cruz Biotechnology,sc-135647,WB/ 1:1000<br>Pol II,Cell Signaling Technology,14958,ChIP/ 5 ug<br>SUZ12,Abcam,ab12073,WB/ 1:1000<br>H3K27me3,Abcam,ab6002,WB/ 1:3000,IHC/ 1:800<br>Ubi,ABclonal,A3207,WB/ 1:1000<br>Anti-Rabbit IgG,Jackson ImmunoResearch,JAC-111-035-003,WB/ 1:10000<br>Anti-Mouse IgG,Jackson ImmunoResearch,JAC-115-035-003,WB/ 1:10000
Validation	All other antibodies in the study were bought commercially. Anti-Actin(AC026), human and mouse, WB, https://abclonal.com.cn/catalog/AC026; Anti-AURKA(14475), human, WB and IP, https://www.cellsignal.cn/products/primary-antibodies/aurora-a-d3e4q-rabbit-mab/14475?site-search-type=Products&N=4294956287&Ntt=aurka&fromPage=plp; Anti-Cad(16617-1-AP), mouse, WB, http://www.ptgcn.com/products/CAD-Antibody-16617-1-AP.htm; Anti-EED(85322), human, WB, https://www.cellsignal.cn/products/primary-antibodies/eed-e4l6e-xp-rabbit-mab/85322?_=1635239606083&Ntt=eed&tahead=true; Anti-EZH1(42088), human, WB, https://www.cellsignal.cn/products/primary-antibodies/ezh1-d7d5d-rabbit-mab/42088?_=1635239764055&Ntt=ezh1&tahead=true; Anti-EZH2(5246),human and mouse,WB, IP and IHC, https://www.cellsignal.cn/products/primary-antibodies/ezh2-d2c9-xp-rabbit-mab/5246?_=1635239825491&Ntt=ezh2&tahead=true; Anti-FBW7a(ab109617), human, WB,https://www.abcam.cn/fbxw7-antibody-ab109617.html; Anti-Flag-tag(F1804), WB and IP, https://www.sigmaaldrich.cn/CN/zh/search/f1804?focus=products&page=1&perPage=30&sort=relevance&term=F1804&type=product; Anti-HA-tag(AE008), WB and IP, https://abclonal.com.cn/catalog/AE008; Anti-HA-tag-HRP(1201381900), WB, https://antibodyregistry.org/search.php?q=AB_390917; Anti-HUWE1(19430-1-AP), human, WB, http://www.ptgcn.com/products/HUWE1-Antibody-19430-1-AP.htm; Anti-Histone H3(ab176842), human, WB, https://www.abcam.cn/histone-h3-antibody-epr16987-nuclear-marker-and-chip-grade-ab176842.html; Anti-Ldha (2012), mouse, WB, https://www.cellsignal.cn/products/primary-antibodies/ldha-antibody/2012?site-search-type=Products&N=4294956287&Ntt=2012&fromPage=plp; Anti-MYC(sc-764), human, WB and IP, https://www.scbt.com/p/c-myc-antibody-n-262?requestFrom=search; Anti-MYC(13987), human, WB, https://www.cellsignal.cn/products/primary-antibodies/c-myc-n-myc-d3n8f-rabbit-mab/13987?site-search-type=Products&N=4294956287&Ntt=13987&fromPage=plp&_requestid=3199466; Anti-myc-tag(AE010), WB and IP, https://abclonal.com.cn/catalog/AE010; Anti-MYCN(sc-53993), human, WB, IP, ChIP and IHC, https://www.scbt.com/p/n-myc-antibody-b8-4-b?requestFrom=search; Anti-p-T58-MYC(sc-135647), human, WB, https://antibodyregistry.org/search.php?q=AB_2148600; Anti-Pol II(14958), human, ChIP, https://www.cellsignal.cn/products/primary-antibodies/rpb1-ntd-d8l4y-rabbit-mab/14958?site-search-type=Products&N=4294956287&Ntt=14958&fromPage=plp&_requestid=3200319; Anti-SUZ12(ab12073), human, WB, https://www.abcam.cn/suz12-antibody-ab12073.html; Anti-H3K27me3(ab 6002), human, WB and IHC, https://www.abcam.cn/histone-h3-tri-methyl-k27-antibody-mabcam-6002-chip-grade-ab6002.html; Anti-Ubi(A3207), human, WB, https://abclonal.com.cn/catalog/A3207; Anti-Rabbit IgG(JAC-111-035-003), WB, https://www.jacksonimmuno.com/catalog/products/111-035-003; Anti-Mouse IgG (JAC-115-035-003), WB, https://www.jacksonimmuno.com/catalog/products/115-035-003.

Eukaryotic cell lines

Policy information about cell lines

Cell line source(s)	293T, NCI-H526, NCI-H2171, NCI-H82 and Daudi cells were purchased from American Type Culture Collection (ATCC). Human neuroblastoma cell lines Kelly, SK-N-BE2, SMS-KAN, SK-N-AS, SH-SY5Y, NBLS, SHEP and BE-2C cells were kindly provided by Drs. John M Maris and Michael D Hogarty (Children's Hospital of Philadelphia, University of Pennsylvania, Philadelphia, PA, USA).
Authentication	Cell lines were authenticated by karyotyping and STR analysis.
Mycoplasma contamination	All cell lines were tested negative for mycoplasma contamination.
Commonly misidentified lines<br>(See ICLAC register)	No commonly misidentified cell lines were used.

Animals and other organisms

Policy information about studies involving animals; ARRIVE guidelines recommended for reporting animal research

Laboratory animals	6-week-old female BALB/c nude mice were purchased from Beijing Vital River Laboratory Animal Technology Co., Ltd. TH-MYCN+/- mice on the 129X1/SvJ genetic background were provided as a courtesy by Prof. William Weiss (University of California-San Francisco, CA, USA). Mice carrying a conditional knockout of Ezh2 alleles Ezh2f/f (Ezh2tm2Sho/J, #022616) and transgenic mice expressing a tamoxifen-inducible cre recombinase under the control of tyrosine hydroxylase (TH) promoter TH-creERT2+/- (Thtm1(cre/Esr1)Nat/J, #008532) were purchased from Jackson Laboratory (JAX).
Wild animals	No wild animals were used in this study.
Field-collected samples	No field-collected samples was used in this study
Ethics oversight	All animal experiments were performed according to animal ethical regulations and with approval from the Institutional Animal Care and Use Committee of Wuhan University.

Note that full information on the approval of the study protocol must also be provided in the manuscript.

ChIP-seq

Data deposition

- [x] Confirm that both raw and final processed data have been deposited in a public database such as GEO.
- [x] Confirm that you have deposited or provided access to graph files (e.g. BED files) for the called peaks.

Data access links<br>*May remain private before publication.*	https://www.ncbi.nlm.nih.gov/geo/query/acc.cgi?acc=GSE181782
Files in database submission	GSM5511325 SK-N-BE-2-Ctrl-MYCN<br>GSM5511326 SK-N-BE-2-EZH2-shRNA-MYCN<br>GSM5511327 SK-N-BE-2-Ctrl-Pol_II<br>GSM5511328 SK-N-BE-2-EZH2-shRNA-Pol_II<br>GSM5511329 SK-N-BE2-Input
Genome browser session<br>(e.g. UCSC)	No longer applicable.

Methodology

Replicates

ChIP-seq was performed in SK-N-BE2 cell line with or without EZH2 depletion using antibodies for BATF3 and pol II once.

Sequencing depth

	Sequenced(reads)	Aligned(reads)
SK-N-BE2-Ctrl-MYCN.bw	13276241	12628591
SK-N-BE2-EZH2-shRNA-MYCN.bw	12906997	11417691
SK-N-BE2-Ctrl-Pol_II.bw	14248289	13266625
SK-N-BE2-EZH2-shRNA-Pol_II.bw	16262538	14882026
SK-N-BE2-Input.bw	1183387	1110750

Antibodies

Name/Catalogue No./Company
anti-MYCN/sc-53993/Santa Cruz Biotechnology
anti-Pol II/14958/Cell Signaling Technology

Peak calling parameters

macs2 callpeak -t SK-N-BE2-Ctrl-MYCN.bam -c SK-N-BE2-Input.bam -f BAM -p 1e-2.

Data quality

Data quality was assessed using macs2 by comparing peak enrichment over input control with a p cutoff value of 1e-2.

Software

ChIP-seq reads were aligned to the human genome (UCSC hg38) with Bowtie2, allowing only uniquely mapping reads with up to two mismatches within the 150 bp reads. The aligned human BAM files were normalized by the normalization factors and converted to bigwig files for visualization in the UCSC Genome Browser. MACS2 (model-based analysis of ChIP-Seq) and Deeptools were used for peak-calling and generation of heatmaps.
